# Editorial: The heart of NAFLD

**DOI:** 10.3389/fmed.2023.1209625

**Published:** 2023-05-22

**Authors:** Nicholas W. S. Chew, Shankar Kannan, Bryan Chong, Yiphan Chin, Mark Muthiah

**Affiliations:** ^1^Yong Loo Lin School of Medicine, National University of Singapore, Singapore, Singapore; ^2^Department of Cardiology, National University Heart Centre, National University Health System, Singapore, Singapore; ^3^Division of Gastroenterology and Hepatology, Department of Medicine, National University Hospital, Singapore, Singapore; ^4^National University Centre for Organ Transplantation, National University Health System, Singapore, Singapore

**Keywords:** non-alcoholic fatty liver disease, cardiovascular disease, coronary artery disease, heart failure, hyperlipidemia, diabetes, hypertension

The silent obesity epidemic has led to the rising global prevalence of non-alcoholic fatty liver disease (NAFLD). This metabolic burden is a growing public health concern as forecast analysis has shown that NAFLD-related disability-adjusted life years will impose a large metabolic burden on the young adult population (1), which will have long-term global implications for the coming decades (1–3). Individuals with NAFLD have a 45% higher risk of fatal and non-fatal cardiovascular events, with cardiovascular-related mortality demonstrated to be the leading cause of death in NAFLD. Those with NAFLD are also at higher risk of incident coronary artery disease, aortic stenosis, heart failure, carotid atherosclerosis, stroke, and atrial fibrillation (4, 5).

The metabolic sequelae of NAFLD, namely insulin resistance, intrahepatic lipid accumulation, inflammation and oxidative stress, increased adiponectin, atherogenic dyslipidemia, the influence on hemostatic-fibrinolytic factors, intestinal microbiome, altered bile acid metabolism, predispose these individuals to atherosclerosis and structural changes to the myocardium. Expectedly, NAFLD is more prevalent in a population with overweight/obesity, although there are geographical variations in its prevalence with the highest amongst those with obesity in America and Europe, but lowest in Southeast Asia (6–11). On the contrary, the prevalence of lean NAFLD in the Asian population is almost doubled that of the Western population (6). Moreover, concomitant metabolic disorders such as hypertension and diabetes mellitus are more common in those with NAFLD, as they share common underlying pathomechanistic pathways and often exist in tandem (12, 13). Concomitant hypertension and diabetes mellitus in individuals with NAFLD exacerbates the cardiovascular risk profile and prognosis (9, 14). In fact, there has been increasing evidence of the genetic cross-talks between NAFLD and cardiovascular diseases (15).

With the increased metabolic milieu in individuals with NAFLD, the global prevalence of coronary heart disease (CHD) has been shown to be 44%. NAFLD patients are at an increased risk of incident CHD compared to those without NAFLD (16), with a step-wise incremental risk of CHD from those with mild hepatic steatosis to those with moderate-severe steatosis. Even in the absence of hypertension, hyperlipidemia and diabetes mellitus, hepatic steatosis and advanced fibrosis were independent predictors of mortality in patients with acute myocardial infarction (17–22). NAFLD is also associated with adverse cardiac remodeling that can lead to impaired systolic and diastolic dysfunction (23, 24). Concomitant metabolic factors and liver disease severity further increase the individual's risk of incident heart failure (25).

The shift from NAFLD to metabolic (26) associated fatty liver disease [MAFLD (Perdomo et al.) (19, 27)] nomenclature also redefines the cardiovascular risk profile of the individual. Carolina et el described that the MAFLD definition accounted for 81% of all NAFLD diagnoses, with MAFLD significantly associated with higher body mass index, hypertension, diabetes mellitus, hyperlipidemia compared to NAFLD (6, 19, 28–30). The use of the MAFLD definition places greater emphasis on the screening and management of concomitant metabolic diseases, and a collaborative effort between cardiologists and hepatologists in the treatment of the chronic liver diseases and cardiovascular risk factors.

There is a need for increased awareness amongst non-hepatologists in the screening of NAFLD in addition to the traditional cardiovascular risk factor testing. A growing body of evidence reveal that readily available non-invasive tests such as the Fibrosis-4 index (FIB-4) have good predictive utility in detecting incident major adverse cardiac events (MACE). Chew et al. reported that a FIB-4 score of ≥2.67 increases the odds of MACE, and is independently associated with 40% increase risk in cardiovascular mortality (27). As traditional cardiovascular risk scoring measures may be inaccurate in NAFLD patients, with alterations in hepatic architecture alterations that can affect the circulatory lipid profile, the integration of hepatic fibrosis screening using the FIB-4 score in traditional CVD risk stratification appears promising (26, 31) although this warrants further validation studies.

With the concerted goal of improving both liver-related and cardiovascular-related outcomes, a multi-disciplinary approach in encouraging sustainable lifestyle measures is paramount. Lifestyle interventions are important to limit energy surplus and enhance weight loss. Antidiabetic medications such as glucagon-like peptide receptor agonists, sodium-glucose cotransporter-2 inhibitors, and pan-peroxisome proliferator-activated receptor-γ (PPAR) agonists (Yagai and Nakamura) have shown improvement in liver-related endpoints in terms of non-alcoholic steatohepatitis (NASH) resolution and fibrosis improvement, as well as concomitant improvement in blood pressure, glycaemic control, atherogenic lipid profile, weight loss, and cardiovascular prognostic endpoints. Yagai and Nakamura provided mechanistic insights into PPARα agonists that act as transcriptional suppressors, which represses target genes on hepatic lipid metabolism, fibrosis and carcinogenesis associated with NAFLD. In addition, bariatric surgery is an effective treatment modality for selected patients with obesity and metabolic syndrome, that can improve both histological characteristics of NASH as well as cardiovascular mortality (32). Lee and colleagues studied endoscopic bariatric and metabolic therapies on weight loss, suggesting promising benefits on weight reduction in intragastric balloon, endoscopic sleeve gastroplasty, and duodeno-jejunal bypass liner, compared to standard medical therapy (Lee et al.). The concerted approach to the reduction of the NAFLD and CVD burden was succinctly consolidated by the study, with the continued medical education program on NAFLD that encompass the best clinical practices tailored to primary care setting, that can improve confidence in the management of NAFLD-related conditions (Papadakis et al.). With the plethora of upcoming NASH therapeutic options (33–35) in the pipeline, treatment strategies in improving both liver-related outcomes and concomitant cardiometabolic profile are promising (32–43).

Whilst hepatology guidelines emphasize the importance of cardiovascular screening in NAFLD, the awareness of the association between NAFLD and CVD amongst cardiologists or in cardiology clinical practice guidelines appears to be lacking. This editorial aims to raise awareness (40) of the liver-heart interface, promoting research and treatment strategies, with both hepatologists and cardiologists working toward the unified goal in addressing the pertinent issues at the heart of NAFLD.

## Author contributions

NC, SK, and MM contributed to conception and design of the study. NC, SK, BC, YC, and MM wrote the manuscript. All authors contributed to manuscript revision, read, and approved the submitted version.
